# THE STUDY OF MUSCLE, MOBILITY AND AGING (SOMMA): LESSONS LEARNED RECRUITING OLDER ADULTS

**DOI:** 10.1093/geroni/igae098.0405

**Published:** 2024-12-31

**Authors:** Kimberly Kennedy, Stephen Kritchevsky, Barbara Nicklas, Anne Newman, Nancy Glynn, Elsa Strotmeyer, Michelle Danielson

**Affiliations:** Wake Forest University School of Medicine, Winston-Salem, North Carolina, United States; Wake Forest University School of Medicine, Wake Forest University School of Medicine, North Carolina, United States; Wake Forest University School of Medicine, Winston-Salem, North Carolina, United States; University of Pittsburgh, University of Pittsburgh, Pennsylvania, United States; University of Pittsburgh School of Public Health, Pittsburgh, Pennsylvania, United States; University of Pittsburgh, Pittsburgh, Pennsylvania, United States; University of Pittsburgh, Pittsburgh, Pennsylvania, United States

## Abstract

SOMMA is a two-site (Wake Forest University School of Medicine [Wake] and University of Pittsburgh [Pitt]) ongoing observational study of 879 participants ≥70 years investigating the role of muscle biology in age-related mobility decline. Those eligible were free from any life-threatening disease, able to walk ≥0.6 m/s (4m) and complete a 400m walk, and safely undergo muscle tissue biopsies and magnetic resonance imaging. Recruitment was from April 2018 to December 2021 and, despite partially spanning the COVID-19 pandemic, exceeded its goal of 875 (Total enrollment: Wake=440; Pitt=439). SOMMA phone screened 4225 persons (Wake=1887; Pitt=2338) with 33.1% (n=1398) eligible for further screening. Advertisement strategies for SOMMA were evaluated and direct mail postcards were most successful and cost-effective; 75% of those phone screened were from direct mailings. At Wake, advertising costs totaled $52k or ~$28/phone screen and $118/enrolled participant. At Pitt, advertising costs totaled $41k or ~$17/phone screen and $93/enrolled participant. Participants eligible after phone screening were invited to attend an in-person information session in which the comprehensive multi-day exam and detailed consent form were reviewed and 4m gait speed was assessed. Sessions were held virtually during pandemic, at which point the gait speed screening was discontinued. A total of 210 information sessions took place (Wake=90; Pitt=120), with an average attendance of ~15 individuals/session and ~80% of those who attended the session were eligible and 63% were ultimately enrolled. SOMMA demonstrated the successful recruitment of a large older adult cohort study for an exam-intensive protocol which included tissue sampling and MR measures.

